# Therapeutic Potential of Pomegranate Extract for Women’s Reproductive Health and Breast Cancer

**DOI:** 10.3390/life14101264

**Published:** 2024-10-03

**Authors:** Jung Yoon Jang, Donghwan Kim, Eunok Im, Nam Deuk Kim

**Affiliations:** 1 Department of Pharmacy, College of Pharmacy, Research Institute for Drug Development, Pusan National University, Busan 46241, Republic of Korea; jungyoon486@pusan.ac.kr; 2 Functional Food Materials Research Group, Korea Food Research Institute, Wanju-gun 55365, Jeollabuk-do, Republic of Korea; kimd@kfri.re.kr

**Keywords:** breast cancer, menopause, polycystic ovary syndrome, pomegranate

## Abstract

Pomegranate extract has potential benefits for women’s reproductive health, including fertility enhancement, menstrual cycle regulation, pregnancy support, and polycystic ovary syndrome (PCOS) treatment. It possesses antioxidant properties, reducing oxidative stress and improving fertility. Pomegranate extract may help regulate hormonal imbalances and promote regular menstrual cycles. The extract’s rich nutrient profile supports placental development and fetal growth and may reduce the risk of preterm birth. Additionally, pomegranate extract shows promise in improving insulin sensitivity and reducing inflammation and oxidative damage in PCOS. Some studies suggest its potential anticancer properties, particularly against breast cancer. However, further research, including human clinical trials, is necessary to establish its effectiveness and safety. The current evidence is limited and primarily based on in vitro studies, animal studies, and clinical trials. This review provides a comprehensive summary of the benefits of pomegranate extract for women’s reproductive health and breast cancer, serving as a reference for future research.

## 1. Introduction

The pomegranate plant (*Punica granatum* L.) is a deciduous shrub that produces fruits. It belongs to the family Lythraceae, specifically the subfamily Punicoideae. Typically, it reaches a height of 5–10 m, equivalent to 16–33 feet [1]. Pomegranate trees are extensively grown in various areas, including the Middle East, Iran, the Caucasus, northern and tropical Africa, the Indian subcontinent, arid parts of Southeast Asia, Central Asia, and the Mediterranean area. They thrive in regions with hot, dry summers and mild winters, making them suitable for cultivation in semi-arid to arid climates. Additionally, pomegranate cultivation has expanded to other parts of the world with similar climatic conditions, such as parts of North and South America, Australia, and southern Europe [2].

The pomegranate fruit comes from the *Punica granatum* tree. Pomegranates typically ripen in the Southern Hemisphere between March and May and in the Northern Hemisphere from September to February [3]. Pomegranates are used in cooking, baking, and preparing smoothies; juice blends; alcoholic beverages, including cocktails and wine; and meal garnishes [4]. Scientifically classified as a berry, pomegranate has been recognized by humanity for >5000 years, with considerable cultural and historical significance [5]. Across various cultures, pomegranates bear symbolic importance, including prosperity, fertility, and abundance [6]. Pomegranates are approximately the size of an apple and have a rounded shape [7]. Encased in a robust, leathery outer skin, their color varies from deep red to yellow [8]. Internally, the fruit contains numerous small, edible seeds surrounded by juicy, ruby-red arils [9]. These arils are not only tasty but are also rich in essential nutrients [10]. Pomegranate have a distinctive combination of sweet and tart flavors, making them a favored option for juices, smoothies, and diverse culinary uses [11]. The taste and physical appearance of salads, yogurt, or desserts are enhanced when the arils of pomegranate are sprinkled on them [12]. In addition to their pleasing flavor, pomegranates are known for their health advantages. Pomegranates have abundant antioxidants, notably punicalagins, and anthocyanins, which protect against oxidative stress and inflammation within the body [13]. The consistent intake of pomegranate is linked to better heart health, a lowered risk of developing certain cancers, and improved immune function [14]. Overall, pomegranates are a delectable and nutritional addition to diet, providing both taste and health benefits [15].

The fruit comprises three components: the tough outer skin (exocarp), pulpy mesocarp, and arils containing seeds, known as kernels [1]. When the exocarp and mesocarp are combined, they form the pericarp, commonly considered part of the pomegranate peel (PP), accounting for nearly 50% of the fruit’s weight. The remaining 50% consists of the arils (40%) and seeds (10%) [16,17]. Singh et al. [18] discovered that PP contains remarkable phytochemicals with medicinal and nutritional significance. PP, along with other parts of the fruit, has been demonstrated to contain nearly 48 phenolic compounds, including polyphenols, flavonoids, ellagitannins, and proanthocyanidins [19,20]. The concentration of these active phytochemicals in pomegranates varies based on the cultivation method, maturity status, geographic region, and processing methods [21]. The juice and peel of pomegranates grown in the desert have a higher phenolic content than those grown in the Mediterranean [22].

Historically, pomegranate has been used as a traditional remedy for conditions such as dysentery, diarrhea, sore throat, hemorrhoids, diabetes, intestinal parasites, and vaginal itching, and a recent review paper on these has been published [4]. Moreover, pomegranate is believed to have tonic properties beneficial for the heart [23]. Furthermore, pomegranate has been recently used for the treatment of a variety of ailments, including Alzheimer’s disease [24], diabetes [25], cancer [17], arthritis [26], obesity [27], male infertility [28], and cardiovascular disorders [29]. In this review, we aimed to discuss the benefits of pomegranate extract on women’s reproductive health, including peri- and post-menopausal symptoms and polycystic ovary syndrome (PCOS), as well as its potential impact on breast cancer. We searched the PubMed, Google Scholar, and ClinicalTrials.gov databases using the following Medical Subject Headings (MeSH) keywords: “breast cancer”, “menopause”, “polycystic ovary syndrome”, and “pomegranate”. The search covered studies up until May 2024, including both preclinical and clinical research. We focused on full-text original articles available in English.

## 2. Pomegranate Extracts

**Figure 1:**
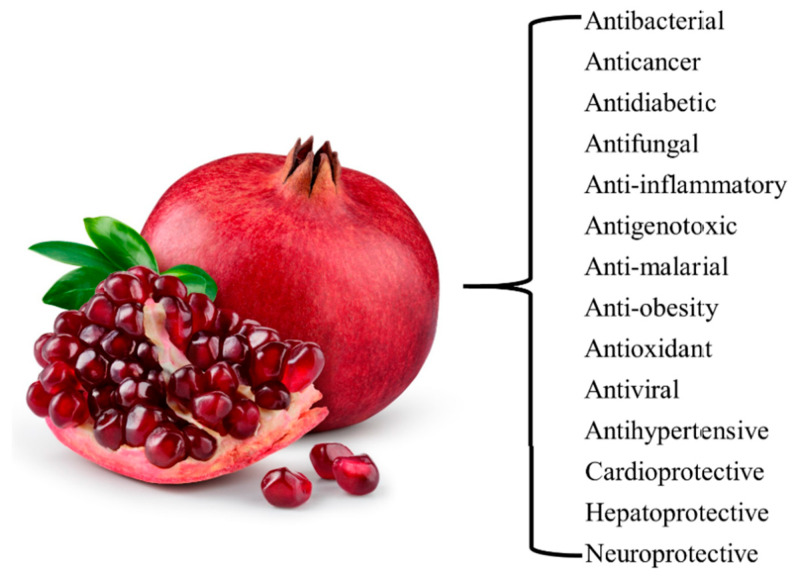
Physiological benefits of pomegranate extract.

Pomegranate extracts reportedly have various physiological benefits, such as antibacterial [30], anticancer [31], antidiabetic [32], antifungal [33], anti-inflammatory [34], antigenotoxic [35], anti-malarial [36], anti-obesity [37], antioxidant [38], antiviral [39], antihypertensive [40], cardioprotective [41], hepatoprotective [42], and neuroprotective [43] (Figure 1).

Pomegranate juice comprises water (85.4%), polyphenols (0.2–1%), sugars (10.6%), and pectins (1.4%) [17]. The vivid color of pomegranate juice is impacted by anthocyanins, and it diminishes during the pressing process [44]. In addition to minerals, including moderately concentrated sodium, selenium, calcium, magnesium, cesium, zinc, and cobalt, the juice contains small amounts of fatty acids, organic acids, sterols, and triterpenoids [45]. Pomegranate juice has a high antioxidant capacity, surpassing other polyphenol-rich beverages and fruit juices, such as red wine, green tea, and various fruit juices [46].

Apart from pomegranate juice, pomegranate seed oil (PSO) can be obtained from different sections of the pomegranate seeds, such as the arils and kernels. Approximately 3% of the weight of a pomegranate is attributed to its seeds, and these seeds contain approximately 12–20% seed oil [29]. Pomegranate seeds are rich in tannins, such as punicalagin and punicalin, as well as tannin precursors, including ellagic acid and gallic acid. They also contain anthocyanins, such as cyanidin, delphinidin, and pelargonidin [47,48]. PSO primarily consists of fatty acids, <95% of its composition [49]. Punic acid, an isomer of linoleic acid unique to pomegranate seeds, accounts for nearly 76% of PSO [50]. Moreover, PSO contains sterols, steroids, and cerebroside. The seed matrix contains varying amounts of isoflavones, lignins, and hydroxybenzoic acids. The seed coat contains organic acids, such as malic, citric, and ascorbic acids [48].

## 3. Nutraceuticals in the Pomegranate Extract and Their Mechanisms of Action

Pomegranates contain various physiologically active compounds with potential health benefits [51]. Pomegranate extract contains phytochemicals (e.g., alkaloids, anthocyanins, flavonoids, lignans, polyphenols, sterols, tannins, terpenes, and terpenoids) that are non-nutritive but have potential health benefits, as well as vitamins, minerals, and fatty acids that provide nutritional and therapeutic benefits. Therefore, we will use the term ‘nutraceuticals’ to refer to all of these components [1,51].

Ellagic acid, present in pomegranate as ellagitannins, is a prevalent bioactive compound known for its antioxidant properties and has been investigated for its possible anticancer properties [52]. Punicalagins, potent antioxidants found in both pomegranate juice and peel, play a significant role in the fruit’s overall antioxidant activity, potentially offering anti-inflammatory and anticancer benefits [53]. Anthocyanins in pomegranate, responsible for its red color, possess antioxidant and anti-inflammatory properties, potentially supporting cardiovascular health [54]. Furthermore, pomegranate contains assorted flavonols, including kaempferol, quercetin, and myricetin, with antioxidant properties, contributing to its anti-inflammatory benefits [55]. Punicic acid, the primary fatty acid in PSO, belongs to the conjugated linolenic acid family and has potential anti-inflammatory and anticancer properties [56]. Pomegranate is rich in vitamins, including vitamins C and B [57]. Vitamin C, an antioxidant, safeguards cells from damage and supports the immune system [58]. The fruit contains essential minerals, including potassium and copper, crucial for bodily functions [57]. In addition to ellagic acid and punicalagin, pomegranate contains other polyphenols, such as catechins and epicatechins, contributing to its overall antioxidant capacity [13].

The mechanisms of anti-inflammatory and anticancer effects of numerous compounds in pomegranate extract, including ellagitannins, anthocyanins, and phenols, have been shown to decrease the expression of cyclooxygenase-2 (COX-2) through the nuclear factor kappa-light-chain-enhancer of activated B cells (NF-κB) and mitogen-activated protein kinase (MAPK) signaling pathways [59,60]. This reduction leads to the lower production of pro-inflammatory prostaglandins and decreased cell proliferation. Additionally, the inhibition of phosphatidylinositol 3-kinases (PI3K), protein kinase B (Akt), or NF-κB itself also contributes to the downregulation of inflammatory gene transcription [59]. Another important transcription factor involved in the expression of pro-inflammatory molecules, known as activation protein 1 (AP-1), can similarly be inhibited by these pomegranate components through the MAPK-mediated phosphorylation of ERK1/2, JNK1, 2, and 3, as well as p38 [61]. Consequently, the transcription of various inflammatory mediators, including tumor necrosis factor (TNF-α), inducible nitric oxide synthase (iNOS), metalloproteinases (MMP), interleukin (IL)-6, and IL-1β, is significantly reduced [62].

**Figure 2:**
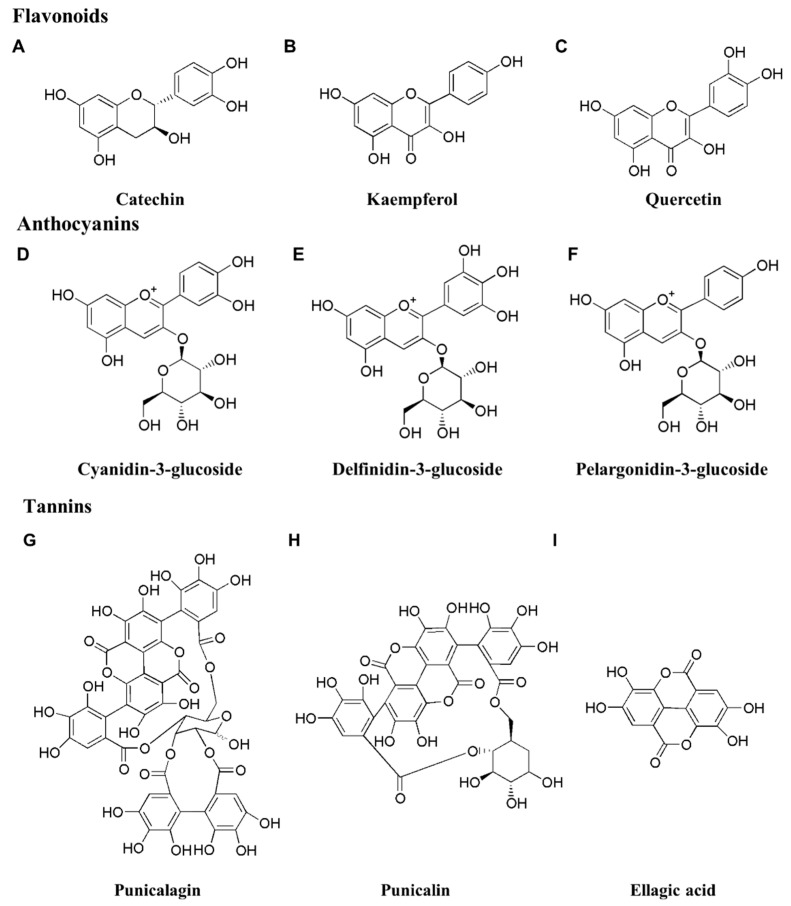

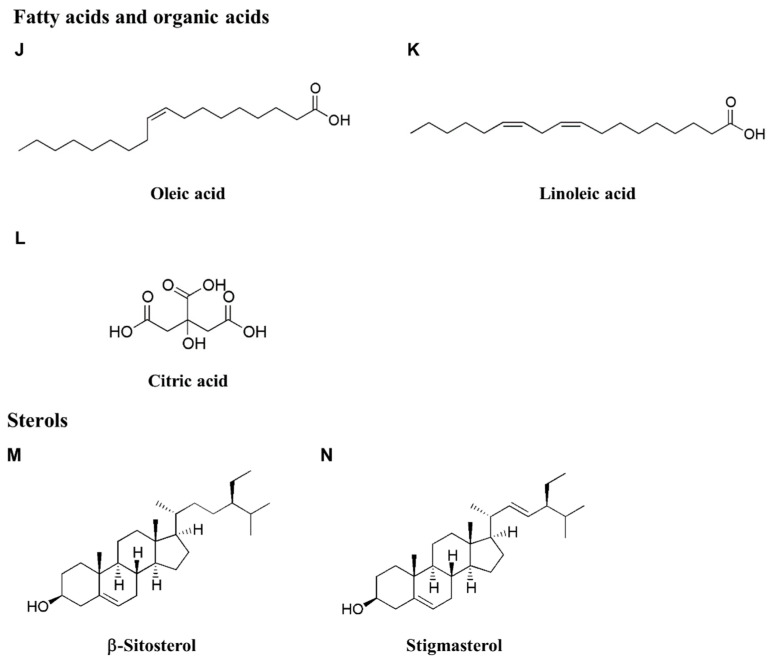
Major nutraceuticals found in pomegranate extract. (**A**–**C**) **Flavonoids:** (**A**) Catechin; (**B**) Kaempferol; (**C**) Quercetin; (**D**–**F**) **Anthocyanins:** (**D**) Cyanidin-3-glucoside; (**E**) Delfinidin-3-glucoside; (**F**) Pelargonidin-3-glucoside; (**G**–**I**) **Tannins:** (**G**) Punicalagin; (**H**) Punicalin; (**I**) Ellagic acid; (**J**–**L**) **Fatty acids and organic acids:** (**J**) Oleic acid; (**K**) Linoleic acid; (**L**) Citric acid; (**M**,**N**) **Sterols:** (**M**) β-Sitosterol; (**N**) Stigmasterol.

We have classified the major nutraceuticals found in pomegranate, and the structures of the identified compounds are illustrated in Figure 2. Additionally, we have classified the nutraceuticals present in pomegranate juice (Table 1) and seed (Table 2) extracts [17,63,64].

### 3.1. Flavonoids

Flavonoids constitute a significant category of natural compounds, are characterized by a polyphenolic structure, and are widely distributed in vegetables, fruits, and certain beverages as secondary plant metabolites [65]. They have crucial health-promoting benefits and find essential applications in medicine, pharmaceuticals, nutraceuticals, and cosmetics [66]. Flavonoids can be classified as anthocyanins, flavones, flavanones, flavonols, chalcones, and isoflavones [67]. These compounds have been found in various parts of the whole fruit, such as the peel, pericarps, leaves, flowers, barks, seeds, and juice [68]. Wang et al. [69] identified numerous prevalent flavonoids, such as prunin, chrysin, catechin, cyanidin, apigenin, luteolin, glucoside, and taxifolin. Figure 2A–C depict the commonly recognized flavonoids in pomegranate.

### 3.2. Anthocyanins

Anthocyanins, active compounds found in pomegranates, are accountable for the fruit’s color from when it begins to ripen until it fully matures [14]. They are made of one or two hexose sugars linked with cyanidin (cyanidin 3-glucoside, cyanidin 3,5-diglucoside, cyanidin 3-rutinoside, cyanidin–pentoside–hexoside, and cyanidin-pentoside), pelargonidin (pelargonidin 3-glucoside and pelargonidin 3,5-diglucoside), and delphinidin (delphinidin 3-glucoside and delphinidin 3,5-diglucoside) [70]. Figure 2D–F depict the common anthocyanins present in pomegranate.

### 3.3. Tannins

Pomegranate, rich in polyphenols, contains tannins, which are present in the seeds and peel [71]. Tannins have various pharmacological properties, including antiviral [72] and antimicrobial [73] properties. Tannins in pomegranate include ellagitannins and gallotannins. Tannins that have been isolated from pomegranate include 3,3′,4′-tri-*O*-methylellagic acid; 2-*O*-galloylpunicalin; 1,2,3-tri-*O*-galloyl-β-4C1-glucose; 3,3′-Di-*O*-methylellagic acid; castalin; castalagin; casuarinin; epicatechin; corilagin; flavogallonic acid; gallagyldilacton; gallagic acid; lagerstannin C; granatin A/B; pedunculagin; punicacortein A, B, C, and D; punicalagin; punicafolin; punicalin α; and β punicatannin [51]. Figure 2G–I depict the tannins commonly found in pomegranate.

### 3.4. Fatty and Organic Acids

Pomegranate contains 83.6% and 16.3% saturated and unsaturated fatty acids, respectively, with the unsaturated fatty acids being a major component of pomegranate seeds [51]. Essential oils, which contain both fatty and organic acids, are known for their various pharmacological benefits, such as anti-parasitic, antimicrobial, pain-relieving, antioxidant, and insect-repellent properties [74]. Volatile compounds and organic oils, such as heneicosanoic, punicic, nonadecanoic, palmitic, stearic, oleic, linolenic, octoic, and linoleic acids, as well as citric acid, have been identified in pomegranates [69,75,76]. Figure 2J–L depict commonly recognized fatty and organic acids in pomegranate.

### 3.5. Sterols

Sterols or phytosterols, classified as natural steroids [77], are the least abundant bioactive compounds found in pomegranate [78]. Their primary role is to decrease cholesterol absorption and low-density lipoprotein cholesterol (LDL-C) in the plasma [77]. Puneeth et al. [79] identified asiatic acid as the main sterol compound, while Wong et al. [80] identified β-sitosterol and stigmasterol. Sex steroids, including estrone, estriolm, and testosterone, are found in pomegranate seeds. Moreover, pomegranates are known to contain several sterol compounds, including campesterol, daucosterol, and sitosteryl-acetate [80]. Figure 2M–N depict sterols commonly found in pomegranate.

## 4. Role of Pomegranate Extract in Alleviating Peri- and Post-Menopausal Symptoms

### 4.1. Peri- and Post-Menopausal Symptoms

The onset of menopause signifies the conclusion of a woman’s reproductive phase, typically happening at approximately 50 years old, though the transition might commence in the late 40s or even earlier [81]. Peri-menopause refers to the period leading up to menopause, while post-menopause refers to the time following the last menstrual period [82]. Many women encounter challenging symptoms throughout peri- and post-menopause, which may involve vasomotor symptoms, such as night sweats and hot flashes (HFs), disruptions in sleep and mood, weight gain, and sexual dysfunction [83,84]. Over 80% of women undergo HFs during menopause, which are marked by sudden, sporadic feelings of warmth, sweating, reddening of the skin, anxiety, and cold sensations lasting from 1 to 5 min [85]. These episodes can be distressing, especially when severe and frequent [86]. Although hot flashes typically diminish with time, around 10–15% of women continue to experience moderate to severe symptoms even a decade or more after reaching menopause [87]. Additionally, menopausal women may face complications, such as osteoporosis, resulting from the loss of post-menopausal bone density due to estrogen deficiency [88]. After menopause, there is a notable increase in the risk of cardiovascular disease (CVD) due to declining estrogen levels. Estrogen and testosterone are pivotal in women’s CVD development, affecting cardiac function, endothelial function, vascular tone, and metabolic syndrome [89]. While menopausal symptoms may not pose a direct threat to life, they impose significant financial burdens on the healthcare system due to their widespread occurrence and adverse impact on quality of life, incurring costs amounting to billions of dollars [90].

### 4.2. Effect of Pomegranate Extract on Peri- and Post-Menopausal Symptoms

Research suggests that pomegranate extract may offer potential health advantages for women in the peri- and post-menopausal stages, positively impacting their overall health [91,92,93,94,95,96]. Research has shown that pomegranate extract is potentially beneficial in alleviating common menopausal symptoms, such as HFs and night sweats [88]. Compounds found in pomegranate that promote bone health may help reduce the heightened risk of osteoporosis and bone loss associated with menopause [92,93]. Pomegranate extract can be advantageous for cardiovascular well-being, particularly relevant during and after menopause. It elevates high-density lipoprotein cholesterol (HDL-C), lowers LDL-C, enhances overall blood cholesterol levels, and decreases the presence of homocysteine, a risk factor for cardiovascular issues. The administration of ellagic acid, an active component of pomegranate, to ovariectomized rats has been reported to increase bone mineral density and trabecular thickness indices, while reducing visceral fat and body weight [91]. Pomegranate extract might be beneficial for maintaining hormonal equilibrium by regulating estrogen levels during menopause-induced hormonal changes [95]. The plentiful antioxidants found in pomegranates have the ability to mitigate oxidative stress and inflammation linked to aging, rendering them particularly beneficial during the menopausal phase [96].

## 5. Role of Pomegranate Extracts in PCOS

### 5.1. PCOS

PCOS is the prevailing endocrine disorder in women, with a prevalence rate of 4–18% among women of reproductive age [97]. PCOS is majorly characterized by the development of enlarged ovaries and numerous small cysts in the external layer of the ovaries [98]. PCOS is associated with an imbalance in the levels of female hormones, namely, estrogen and progesterone, leading to an elevation in male hormones (androgens). This imbalance results in symptoms such as irregular menstrual cycles, hair loss, acne, and fertility issues [99]. Many women diagnosed with PCOS encounter insulin resistance, a condition where the body’s cells respond poorly to insulin [100]. This resilience can lead to increased insulin levels, which can contribute to weight gain and a heightened vulnerability to type 2 diabetes [101]. Additionally, PCOS is linked to metabolic irregularities, including dyslipidemia (abnormal lipid levels) and obesity, heightening the likelihood of developing CVD [102]. The exact origin of PCOS remains partially unclear, but it is believed to stem from a combination of genetic predisposition and environmental influences [103]. The diagnosis of PCOS commonly relies on a combination of clinical indications, symptoms, and particular criteria, such as the Rotterdam criteria [104]. The emphasis in treatment lies in managing the symptoms and minimizing correlated health risks through lifestyle adjustments, including engaging in regular physical activity, maintaining a balanced diet, and managing weight [100]. Hormonal birth control pills may be recommended to regulate menstrual patterns and androgen levels [98]. To effectively treat PCOS, a comprehensive approach that considers both hormonal and metabolic dimensions is necessary [105].

### 5.2. Effect of Pomegranate Extract on PCOS

Although there have not been numerous studies on the efficacy of pomegranate extract in PCOS, some in vivo research has reported its effectiveness [106,107]. Hossein et al. [106] reported a significant increase in the concentrations of estrogen, testosterone, and androstenedione in the estradiol valerate-induced PCOS model compared with the control group, and they also reported that the elevated hormone levels in the PCOS model treated with pomegranate juice extract exhibited a significant decrease. Therefore, they recommended the consumption of pomegranate juice extract to improve the complex symptoms associated with PCOS. Ibrahim et al. [107] observed a notable elevation in serum levels of luteinizing hormone (LH), follicle-stimulating hormone (FSH), estradiol, testosterone, and tissue malondialdehyde (MDA) in the PCOS group and histological alterations in the endometrial tissues. There was a considerable increase in the amount of collagen fibers in the endometrium, a noticeable increase in the expression of Ki67 and androgen receptor through immunohistochemistry, and a significant reduction in the average count of pinopodes. The concurrent application of pomegranate juice extract successfully normalized the levels of the investigated histological, biochemical, and immunohistochemical parameters. The endometrial histological changes associated with PCOS were reversed by pomegranate juice extract. Moreover, this effect can be attributed to its polyphenolic content, which has anti-inflammatory, antioxidant, antiproliferative, antifibrotic, and anti-androgenic effects.

## 6. Role of Pomegranate Extracts in Breast Cancer

### 6.1. Breast Cancer

Breast cancer ranks as the second highest cause of cancer-related fatalities among women and has recently become the most prevalent type of cancer in the United States [108]. The widely recognized risk factors for developing breast cancer include getting older, having a family history of the disease, starting menstruation early, experiencing menopause later, the prolonged use of estrogen replacement therapy, and having children at a later age [64]. There is a consensus that steroid hormones, especially estrogen, exert a substantial influence on the development of breast cancer [109]. Breast cancer treatment depends on various factors, including tumor type, size, stage, and the overall health of the patient. Frequently employed approaches to treat breast cancer encompass surgery, hormone therapy, chemotherapy, radiation therapy, and immunotherapy [110]. The mortality rate associated with breast cancer mortality rate is steadily increasing, recently prompting researchers to emphasize prevention as a key management strategy for this disease [111]. For centuries, the significance of natural substances has been acknowledged, with various factions advocating for the use of plant-derived compounds to prevent or treat diseases like breast cancer [112]. These naturally occurring compounds, referred to as nutraceuticals, have garnered attention for their beneficial effects, particularly in plant-derived foods containing polyphenolic compounds [113].

### 6.2. Effect of Pomegranate Extract on Breast Cancer

Pomegranate extract has potential anticancer properties, especially against breast cancer [17,114]. Abundant in polyphenols, such as ellagic acid and punicalagin, pomegranate has exhibited cell antiproliferative, anti-estrogenic, anti-inflammatory, and anti-angiogenic properties, indicating its potential to hinder the development and metastasis of breast cancer cells [115]. Polyphenols found in pomegranate juice, pericarp (peel), and seed oil inhibit aromatase and 17β-hydroxysteroid dehydrogenase, enzymes responsible for converting androgens into estrogens, thereby preventing the development of breast cancer [116]. Ellagitannin-derived compounds, such as ellagic acid, gallagic acid, and urolithins A and B, exhibit aromatase inhibitory effects. In particular, urolithin B acts as the most potent aromatase inhibitor, playing a crucial role in suppressing the proliferation of breast cancer cells [117]. Punicic acid, a type of polyunsaturated fatty acid present in pomegranate seed oil, inhibits cell growth and induces apoptosis in breast cancer cell lines (MDA-MB-231, MDA-ER-7) independently of estrogen dependency [118]. Pomegranate extract is capable of causing cell cycle arrest, a vital mechanism for preventing the unchecked growth of cancer cells. Additionally, it may trigger apoptosis, a programmed cell death process, in breast cancer cells, thereby contributing to the removal of impaired or abnormal cells [119,120]. Furthermore, its anti-estrogenic properties might be advantageous in the treatment of hormone-dependent breast cancer [64]. The polyphenols in pomegranates can inhibit pro-inflammatory cytokines (IL-2, IL-4, IL-6, IL-12, IL-17, interferon-gamma inducible protein-10, monocyte chemoattractant protein-1, macrophage inflammatory protein (MIP)-1α, MIP-1β, and tissue necrosis factor-α) and suppress inflammation. Additionally, it may help prevent angiogenesis by reducing the levels of vascular endothelial growth factor, a cytokine involved in the growth and formation of blood vessels, and promoting the migration inhibitory factor, a protein associated with cell movement inhibition. Inhibiting angiogenesis can limit tumor growth [121,122]. Pomegranate extracts have potential synergistic effects with existing breast cancer treatments, such as chemotherapy and hormone therapy, thereby improving their effectiveness [85].

## 7. Clinical Effects of Pomegranate Extract on Women’s Health

Several clinical studies of the effects of pomegranate extract in peri- and post-menopausal symptoms, PCOS, and breast cancer have been reported, and they are summarized in Table 3.

### 7.1. Clinical Effects of Pomegranate Extract against Peri- and Post-Menopausal Symptoms

Clinical trials of pomegranate extract for the treatment of peri- and post-menopausal symptoms have been reported. Kim et al. [123] investigated the effect of pomegranate extract in 58 menopausal women. The participants were randomly assigned to two groups: one group received pomegranate extracts at a dosage of 1.5 g per day, while the other group took a placebo orally for 8 weeks. The group that received pomegranate extract exhibited a significant reduction in HF score, HF visual analog scale (VAS), HF duration, and sweating VAS, indicating a notable decrease in patients’ HFs and sweating. Furthermore, the menopausal rating scales and Kupperman index scores significantly decreased in the group that consumed pomegranate extract. This outcome indicates a potential benefit of pomegranate extracts for women experiencing menopause.

Huber et al. [124] investigated the safety and effectiveness of PSO in patients with menopausal symptoms. Over the course of 46 months, 78 patients experiencing menopausal symptoms consumed 1000 mg of PSO daily, taking 2 capsules per day. The individuals demonstrated notable improvements across all aspects of the menopausal rating scale, particularly urogenital symptoms (dry vagina).

### 7.2. Clinical Effects of Pomegranate Extract on PCOS

Multiple clinical studies have documented the potential impacts of pomegranate extracts on PCOS. Esmaeilinezhad et al. [130] reported the effects of synbiotic pomegranate juice (SPJ) on glycemic indices, sex hormone profiles, and anthropometric indices in PCOS. Ninety-two patients with PCOS were administered 2 L per week of SPJ, pomegranate juice (PJ), and synbiotic beverage (SB) for 8 weeks. The control group received 2 L of a placebo beverage (PB) per week. SPJ contained pomegranate juice with inulin and lactobacillus, SB consisted of water, inulin, lactobacillus, and pomegranate flavoring, and PB contained water and pomegranate flavoring. Substantial alterations were observed in insulin resistance within the SPJ and SB groups, with a notable increase in insulin sensitivity. Insulin levels changed significantly in both the SPJ and SB groups. In addition, body mass index (BMI), weight, and waist circumference were significantly reduced in the SPJ and SB groups. Furthermore, a significant decrease in testosterone levels was observed in both the SPJ and SB groups. Conversely, there were no significant changes in the levels of LH, fasting plasma glucose, and FSH across any of the groups. The findings indicate that the innovative beverage SPJ may have the potential to enhance insulin levels, reduce insulin resistance, regulate testosterone levels, aid in weight management, lower BMI, and decrease waist circumference among individuals diagnosed with PCOS.

Abedini et al. [132] studied the impact of concentrated pomegranate juice (CPJ), a different type of pomegranate extract, on CVD risk factors among women diagnosed with PCOS. In their trial, which included 44 women aged 18–40 with PCOS and a BMI of 25 kg/m^2^ or higher, the intervention group consumed 45 mL CPJ daily with 180 mL of water. Compared with the control group, CPJ significantly reduced systolic and diastolic blood pressure, serum triglyceride (TG) levels, and the TG/HDL-C ratio. These discoveries indicate that CPJ has positive effects on blood pressure, serum levels of TG and HDL-C, and the TG/HDL-C ratio among women with PCOS.

Based on the study conducted by Kazemi et al. [134], ellagic acid derived from pomegranate was found to have a profound impact on PCOS. The research demonstrated that ellagic acid significantly reduces insulin resistance, alleviates oxidative stress, and aids in the regulation of sex hormone levels. These findings suggest that ellagic acid could serve as a beneficial supplement in the treatment of PCOS by improving metabolic and hormonal imbalances.

### 7.3. Clinical Effects of Pomegranate Extract on Breast Cancer

Pomegranate extract and polyphenols have been thoroughly investigated for their potential to combat cancer, particularly in studies focusing on breast cancer, conducted both in vitro and in vivo [135,136,137,138]. However, presently, there is only one reported clinical study [114]. This limited number of clinical studies may be attributed to the availability of various anticancer agents for breast cancer treatment. Kapoor et al. [114] examined the impact of drinking pomegranate juice on hormonal indicators linked to the risk of breast cancer. Sixty-four post-menopausal women, who were in good health, were randomly assigned to consume either 8 ounces (236.6 mL) of 100% commercial pomegranate juice (as the intervention group) or apple juice (as the control group) for a duration of 3 weeks. Overall, individuals consuming pomegranate juice did not experience significant reductions in serum sex hormone levels or sex hormone-binding globulin compared to those in the control group. Yet, upon examining a subset comprising 38 women of average weight, those who consumed pomegranate juice exhibited a notable reduction in estrogen and testosterone levels in contrast to the control group.

## 8. Conclusions

Pomegranate extract is an abundant reservoir of diverse compounds known for their advantageous physiological effects, especially antioxidant and anti-inflammatory properties. In women’s reproductive health, pomegranate extract contributes to bone health and prevents osteoporosis in peri- and post-menopausal women through the activities of certain compounds, such as antioxidants and ellagic acid. Additionally, in peri- and post-menopausal women, pomegranate extract causes improvements in cholesterol levels and blood pressure, reducing the risk of heart diseases, such as atherosclerosis. Its anti-inflammatory properties can be used in treating symptoms related to hormonal changes during menopause. Pomegranate extract is beneficial in women with PCOS as it improves insulin sensitivity. Its anti-inflammatory effects can assist in managing chronic inflammation associated with PCOS. Pomegranate extract reportedly influences hormonal balance, particularly by regulating reproductive hormones in women with PCOS. Pomegranate extract exhibits potent antioxidant and anti-inflammatory effects, with specific compounds like ellagic acid having anticancer properties. Pomegranate extract may influence hormonal receptors in breast cancer cells, particularly affecting hormone-sensitive breast cancer. So far, the efficacy of pomegranate extract in peri- and post-menopausal women, patients with PCOS, and patients with breast cancer has been observed clinically. However, more clinical studies are needed to determine the appropriate formulations and dosages for using pomegranate extracts to prevent or treat these conditions. Additionally, while this review paper provides a comprehensive summary of the efficacy and clinical effects of pomegranate extract on women’s health, including peri- and post-menopausal symptoms, PCOS, and breast cancer, there is a lack of reported studies on the molecular mechanisms, which is a notable deficiency. Therefore, further research focusing on the specific molecular mechanisms is essential.

## Figures and Tables

**Table 1:** Nutraceuticals in pomegranate juice.

Chemical Class	Nutraceuticals
Amino acids	Aspartic acid, Glutamic acid, Methionine, Proline, Valine
Anthocyanins	Cyanidin-3-*O*-glucoside, Cyanidin-3,5-di-*O*-glucosdie, Delphinidin-3-*O*-glucoside, Delphinidin-3,5-di-*O*-glucoside, Pelargonidin-3-*O*-glucoside, Pelargonidin-3,5-di-*O*-glucoside
Ellagitannins	Casuarinin, Corilagin, Gallagyldilacton, Punicalagin, Punicalin
Flavan-3-ols	Catechin, Epicatechin, Epigallocatechin-3-gallate
Flavonols	Quercetin, Isoquercetin, Rutin
Hydroxybenzoic acid/Hydroxycinnamic acid	Caffeic acid, Chlorogenic acid, Ellagic acid, Gallic acid, Quinic acid
Indoleamines	Melatonin, Serotonin, Tryptamine
Organic acids	Ascorbic acid, Citric acid, Fumaric acid, Malic acid, Succinic acid, Tartaric acid
Sugars	Glucose, Fructose, Sucrose

**Table 2:** Nutraceuticals in pomegranate seeds.

Chemical Class	Nutraceuticals
Conjugated fatty acids	Punicic acid
Non-conjugated fatty acids	Linoleic acid, Oleic acid, Palmitic acid, Stearic acid
Hydroxybenzoic acid	3,3′-Di-*O*-methylellagic acid, Ellagic acid, 3,3′-4′-Tri-*O*-methylellagic acid
Isoflavones	Daidzein, Genistein
Lignins	Coniferyl-9-*O*-[β-dapiofuranosyl(1→6)-*O*-β-D-glucopyranoide, Icariside D1, Phenylethyl rutinoside, Sinapyl-9-*O*-[βD-apiofuranosyl(1→6)-*O*-β-D-glucopyranoide
Sterols and steroids	Camesterol, Cholesterol, 17-α-Estradiol, Estriol, Estrone, β-Sitosterol, Stigmasterol, Testosterone
Tocopherols	γ-Tocopherol
Triterpenes	Oleanolic acid, Ursolic acid

**Table 3:** Clinical effects of pomegranate extract on women’s health conditions.

Conditions	Interventions	Dosage/Duration	Brief Summary	Outcomes	Refs.
***Menopausal Symptoms***					
MS women (experience of menstrual irregularities lasting for more than 3 months or amenorrhea persisting for over 1 year post-menopause)	PE, placebo	125 mg of PE per 500 mg tablet, 12 tablets per day (=1.5 g of PE per day)/8 weeks	Effects of PE on MS women	Decrease in HF score, HF VAS, sweating VAS, HF duration, MRS, KI, MSs frequency, and MENQOL	[123]
MS women (mean duration of MSs of 46 months)	PSO, placebo	1000 mg of PSO per day in 2 capsules/8 weeks	Investigating the safety and effectiveness of PSO for MSs	Reduction in MRS symptoms (HFs); improvement in urogenital tract symptoms (dry vagina)	[124]
Post-MS women (12 months of amenorrhea)	PSO, placebo	Two doses of 30 mg PSO per day/12 weeks	Investigating the potential effects of PSO on MSs	Decrease in the score of the menopause rating scale II, the number of HFs, and the sum score of vegetative somatic symptoms; improvement in sleeping disorders	[125]
MS women	Pomegranate supplement, placebo	3 mL three times per day/4 weeks	Effects of pomegranate supplement on MSs and quality of life in menopausal women	Decrease in modified-KI and MENQOL	[126]
Post-MS women with type 2 diabetes	CW, PE, TW, TPE	150 mL of PE per day/6 weeks	Effects of aerobic exercise and PE on antioxidant markers in post-MS women with type 2 diabetes	Increase in GPX, SOD, GSH, and TAC; the highest levels of the antioxidants in TPE	[127]
Osteopenic women	2 PE capsules, 2 placebo capsules	2 PE capsules per day/6 month	Changes in biomarkers related to bone absorption and formation in post-MS women	Urinary NTX and serum P1NP in post-MS women (completed; no study results published)	[128]
Pre-MS women	PE, placebo	2 mL of PE three times per day/10 days during the 3 menstrual cycles (from 7 days before to 3 days after the estimated onset of menstruation)	Effect of pomegranate supplementation on symptom severity in pre-MS women	Pre-MS scale (recruiting)	[129]
***PCOS***					
PCOS patients	SPJ, PJ, SB, PB	2 L of PJ, SB, SPJ, PB per week/8 weeks	Effect of SPJ on glycemic, sex hormone profile, and anthropometric indices in PCOS	Decrease in insulin resistance, FBS, testosterone, BMI, waist, and hip circumference; increase in insulin sensitivity	[130]
PCOS patients	SPJ, PJ, SB, PB	300 mL of PJ, SB, SPJ, PB per day/8 weeks	The effect of SPJ on cardiovascular risk factors in PCOS patients	Decrease in TC, LDL-C, MDA, hs-CRP, and blood pressure; increase in HDL-C and TAC	[131]
Women with PCOS (18–40 years and BMI of ≥25 kg/m^2^)	CPJ, placebo	45 mL of CPJ in combination with 180 mL water per day/8 weeks	The effect of CPJ consumption on risk factors of cardiovascular diseases in women with PCOS	Decrease in systolic and diastolic blood pressure, TG levels, and TG/HDL-C ratio; increase in LDL-C and HDL-C	[132]
Women with PCOS (18–40 years and BMI of ≥25 kg/m^2^)	CPJ, placebo	45 mL of CPJ in combination with 180 mL water per day/8 weeks	The effect of CPJ on biomarkers of inflammation, oxidative stress, and sex hormones in overweight and obese women with PCOS	Decrease in serum testosterone levels	[133]
***Breast Cancer***					
Healthy post-MS women	100% commercial PJ, 100% apple juice (control)	8 ounces (4 ounces in the morning and 4 ounces in the early evening) per day/3 weeks	Effects of PJ on hormonal biomarkers of breast cancer risk	Decrease in estrone and testosterone levels (normal-weight women)	[114]

BMI, body mass index; CPJ, concentrated pomegranate juice; CW, control water; FBS, fasting blood sugar; GPX, glutathione peroxidase; GSH, plasma glutathione; HDL-C, high-density lipoprotein cholesterol; HFs, hot flashes; hs-CRP, high sensitive C-reactive protein; KI, Kupperman index; LDL-C, low-density lipoprotein cholesterol; MDA, malondialdehyde; MENQOL, menopause-specific quality of life; MRS, menopausal rating scale; MSs, menopausal symptoms; NTX, N-terminal telopeptide; PB, placebo beverage; PCOS, polycystic ovarian syndrome; PE, pomegranate extract; P1NP, procollagen type 1 amino-terminal propeptide; PJ, pomegranate juice; PSO, pomegranate seed oil; SB, synbiotic beverage; SOD, superoxide dismutase; SPJ, synbiotic pomegranate juice; TAC, total antioxidant capacity; TC, total cholesterol; TG, serum triglyceride; TPE, training-pomegranate extract; TW, training-water; VAS, visual analog scale.

## Data Availability

Data presented in this study are available on request from the corresponding author.

## References

[1] Montefusco A., Durante M., Migoni D., De Caroli M., Ilahy R., Pék Z., Helyes L., Fanizzi F.P., Mita G., Piro G. (2021). Analysis of the phytochemical composition of pomegranate fruit juices, peels and kernels: A comparative study on four cultivars grown in southern Italy. Plants.

[2] Bar-Ya’akov I., Tian L., Amir R., Holland D. (2019). Primary metabolites, anthocyanins, and hydrolyzable tannins in the pomegranate fruit. Front. Plant Sci..

[3] Kosseva M.R., Joshi V.K., Panesar P.S. (2016). Science and Technology of Fruit Wine Production.

[4] Eghbali S., Askari S.F., Avan R., Sahebkar A. (2021). Therapeutic effects of *Punica granatum* (pomegranate): An updated review of clinical trials. J. Nutr. Metab..

[5] Paris H.S. (2015). Origin and emergence of the sweet dessert watermelon, *Citrullus lanatus*. Ann. Bot..

[6] Langley P. (2000). Why a pomegranate?. BMJ.

[7] Wetzstein H.Y., Zhang Z., Ravid N., Wetzstein M.E. (2011). Characterization of attributes related to fruit size in pomegranate. HortScience.

[8] Kumar N.V., Godara A., Mirza A. (2020). Characteristics of flowering and fruiting description of pomegranate (*Punica granatum* L.). J. Am. Soc. Hortic. Sci..

[9] Kahramanoglu I., Usanmaz S. (2016). Pomegranate Production and Marketing.

[10] Amos Fawole O., Linus Opara U. (2012). Composition of trace and major minerals in different parts of pomegranate (*Punica granatum*) fruit cultivars. BFJ.

[11] Mayuoni-Kirshinbaum L., Porat R. (2014). Agriculture. The flavor of pomegranate fruit: A review. J. Sci. Food Agric..

[12] Buyuran F. (2015). Pomegranates and Saffron: A Culinary Journey to Azerbaijan.

[13] Saparbekova A.A., Kantureyeva G.O., Kudasova D.E., Konarbayeva Z.K., Latif A.S. (2023). Potential of phenolic compounds from pomegranate (*Punica granatum* L.) by-product with significant antioxidant and therapeutic effects: A narrative review. Saudi J. Bio. Sci..

[14] de Oliveira F.L., Arruda T.Y.P., da Silva Lima R., Casarotti S.N., Morzelle M.C. (2020). Pomegranate as a natural source of phenolic antioxidants: A review. J. Food Bioact..

[15] Viuda-Martos M., Fernández-López J., Pérez-Álvarez J.A. (2010). Pomegranate and its many functional components as related to human health: A review. Compr. Rev. Food Sci. Food Saf..

[16] Lampakis D., Skenderidis P., Leontopoulos S. (2021). Technologies and extraction methods of polyphenolic compounds derived from pomegranate (*Punica granatum*) peels. A mini review. Processes.

[17] Moga M.A., Dimienescu O.G., Bălan A., Dima L., Toma S.I., Bîgiu N.F., Blidaru A. (2021). Pharmacological and therapeutic properties of *Punica granatum* phytochemicals: Possible roles in breast cancer. Molecules.

[18] Singh B., Singh J.P., Kaur A., Singh N. (2018). Phenolic compounds as beneficial phytochemicals in pomegranate (*Punica granatum* L.) peel: A review. Food Chem..

[19] Magangana T.P., Makunga N.P., Fawole O.A., Opara U.L. (2020). Processing factors affecting the phytochemical and nutritional properties of pomegranate (*Punica granatum* L.) peel waste: A review. Molecules.

[20] Milošević M., Vulić J., Kukrić Z., Lazić B., Četojević-Simin D., Čanadanović-Brunet J. (2023). Polyphenolic composition, antioxidant and antiproliferative activity of edible and inedible parts of cultivated and wild pomegranate (*Punica granatum* L.). Food Technol. Biotechnol..

[21] Guerrero-Solano J.A., Bautista M., Espinosa-Juárez J.V., Moreno-Rocha L.A., Betanzos-Cabrera G., Salanță L.C., De la O Arciniega M., Olvera-Hernández E.G., Jaramillo-Morales O.A. (2022). Differential antinociceptive efficacy of peel extracts and lyophilized juices of three varieties of Mexican pomegranate (*Punica granatum* L.) in the formalin test. Plants.

[22] Schwartz E., Tzulker R., Glazer I., Bar-Ya’akov I., Wiesman Z., Tripler E., Bar-Ilan I., Fromm H., Borochov-Neori H., Holland D. (2009). Environmental conditions affect the color, taste, and antioxidant capacity of 11 pomegranate accessions’ fruits. J. Agric. Food Chem..

[23] Mohammad S.M., Kashani H.H. (2012). Chemical composition of the plant *Punica granatum* L. (pomegranate) and its effect on heart and cancer. J. Med. Plants Res..

[24] Almuhayawi M.S., Ramadan W.S., Harakeh S., Al Jaouni S.K., Bharali D.J., Mousa S.A., Almuhayawi S.M. (2020). The potential role of pomegranate and its nano-formulations on cerebral neurons in aluminum chloride induced Alzheimer rat model. Saudi J. Biol. Sci..

[25] Faddladdeen K.A., Ojaimi A.A. (2019). Protective effect of pomegranate (*Punica granatum*) extract against diabetic changes in adult male rat liver: Histological study. J. Microsc. Ultrastruct..

[26] Basu A., Schell J., Scofield R.H. (2018). Dietary fruits and arthritis. Food Funct..

[27] Michicotl-Meneses M.M., Thompson-Bonilla M.d.R., Reyes-López C.A., García-Pérez B.E., López-Tenorio I.I., Ordaz-Pichardo C., Jaramillo-Flores M.E. (2021). Inflammation markers in adipose tissue and cardiovascular risk reduction by pomegranate juice in obesity induced by a hypercaloric diet in Wistar rats. Nutrients.

[28] Alshinnawy A., Elsayed W., Taha A., Sayed A., Salem A. (2020). *Astragalus membranaceus* and *Punica granatum* alleviate infertility and kidney dysfunction induced by aging in male rats. Turk. J. Biol..

[29] Wang J., Sun M., Yu J., Wang J., Cui Q. (2024). Pomegranate seeds: A comprehensive review of traditional uses, chemical composition, and pharmacological properties. Front. Pharmacol..

[30] Kupnik K., Primožič M., Vasić K., Knez Ž., Leitgeb M. (2021). A Comprehensive study of the antibacterial activity of bioactive juice and extracts from pomegranate (*Punica granatum* L.) peels and seeds. Plants.

[31] Usha T., Middha S.K., Sidhalinghamurthy K.R. (2020). Pomegranate peel and its anticancer activity: A mechanism-based review. Plant-Derived Bioactives.

[32] Amri Z., Ben Khedher M.R., Zaibi M.S., Kharroubi W., Turki M., Ayadi F., Hammami M. (2020). Anti-diabetic effects of pomegranate extracts in long-term high fructose-fat fed rats. Clin. Phytoscience.

[33] Jahani M., Pira M., Aminifard M.H. (2020). Antifungal effects of essential oils against Aspergillus niger in vitro and in vivo on pomegranate (*Punica granatum*) fruits. Sci. Hortic..

[34] Stefanou V., Papatheodorou S., Tsakni A., Lougovois V., Talelli A., Panourgias G., Dariatos A., Tsaknis I. (2020). Anti-inflammatory properties of pomegranate. Int. J. Adv. Res. Microbiol. Immunol..

[35] Uluman E., Kilicle P.A. (2020). The investigation of the possible antigenotoxic in vivo effects of pomegranate (*Punica granatum* L.) peel extract on mitomycin-C genotoxicity. Turk. J. Vet. Anim. Sci..

[36] Raj A.A.S., Rekhaa V.S., Suvedha P., Priya N.R. (2014). Pomegranate: Constituents, biological properties, therapeutic applications and its safety—A Review. Int. J. Adv. Life Sci..

[37] Mayasankaravalli C., Deepika K., Esther Lydia D., Agada R., Thagriki D., Govindasamy C., Chinnadurai V., Othman Gatar O.M., Khusro A., Kim Y.O. (2020). Profiling the phyto-constituents of *Punica granatum* fruits peel extract and accessing its in-vitro antioxidant, anti-diabetic, anti-obesity, and angiotensin-converting enzyme inhibitory properties. Saudi J. Biol. Sci..

[38] Jalili S., Tabatabee Naini A., Ashrafi M., Aminlari M. (2020). Antioxidant activity of pericarp extract from different varieties of pomegranate fruit. J. Agric. Sci. Technol..

[39] Salles T.S., Meneses M.D.F., Caldas L.A., Sá-Guimarães T.E., de Oliveira D.M., Ventura J.A., Azevedo R.C., Kuster R.M., Soares M.R., Ferreira D.F. (2021). Virucidal and antiviral activities of pomegranate (*Punica granatum*) extract against the mosquito-borne Mayaro virus. Parasites Vectors.

[40] Hernández-Corroto E., Plaza M., Marina M.L., García M.C. (2020). Sustainable extraction of proteins and bioactive substances from pomegranate peel (*Punica granatum* L.) using pressurized liquids and deep eutectic solvents. Innov. Food Sci. Emerg. Technol..

[41] Niewiadomska J., Kasztura M., Janus I., Chełmecka E., Stygar D.M., Frydrychowski P., Wojdyło A., Noszczyk-Nowak A. (2023). *Punica granatum* L. Extract shows cardioprotective effects measured by oxidative stress markers and biomarkers of heart failure in an animal model of metabolic syndrome. Antioxidants.

[42] Murtaza S., Khan J.A., Aslam B., Faisal M.N. (2021). Pomegranate peel extract and quercetin possess antioxidant and hepatoprotective activity against concanavalin a-induced liver injury in mice. Pak. Vet. J..

[43] Emami Kazemabad M.J., Asgari Toni S., Tizro N., Dadkhah P.A., Amani H., Akhavan Rezayat S., Sheikh Z., Mohammadi M., Alijanzadeh D., Alimohammadi F. (2022). Pharmacotherapeutic potential of pomegranate in age-related neurological disorders. Front. Aging Neurosci..

[44] Robert P., Gorena T., Romero N., Sepulveda E., Chavez J., Saenz C. (2010). Encapsulation of polyphenols and anthocyanins from pomegranate (*Punica granatum*) by spray drying. Int. J. Food Sci. Technol..

[45] Waheed S., Siddique N., Rahman A., Zaidi J., Ahmad S. (2004). INAA for dietary assessment of essential and other trace elements in fourteen fruits harvested and consumed in Pakistan. J. Radioanal. Nucl. Chem..

[46] Aloqbi A., Omar U., Yousr M., Grace M., Lila M.A., Howell N. (2016). Antioxidant activity of pomegranate juice and punicalagin. Nat. Sci..

[47] Afaq F., Saleem M., Krueger C.G., Reed J.D., Mukhtar H. (2005). Anthocyanin-and hydrolyzable tannin-rich pomegranate fruit extract modulates MAPK and NF-kappaB pathways and inhibits skin tumorigenesis in CD-1 mice. Int. J. Cancer.

[48] Walid E., Hedia H., Nizar T., Yassine Y., Nizar N., Ali F. (2012). Total phenolic contents and antioxidant activities of pomegranate peel, seed, leaf and flower. J. Med. Plant Res..

[49] Lansky E.P., Newman R.A. (2007). *Punica granatum* (pomegranate) and its potential for prevention and treatment of inflammation and cancer. J. Ethnopharmacol..

[50] Kohno H., Suzuki R., Yasui Y., Hosokawa M., Miyashita K., Tanaka T. (2004). Pomegranate seed oil rich in conjugated linolenic acid suppresses chemically induced colon carcinogenesis in rats. Cancer Sci..

[51] Maphetu N., Unuofin J.O., Masuku N.P., Olisah C., Lebelo S.L. (2022). Pharmacotherapy. Medicinal uses, pharmacological activities, phytochemistry, and the molecular mechanisms of *Punica granatum* L. (pomegranate) plant extracts: A review. Biomed. Pharmacother..

[52] Usta C., Ozdemir S., Schiariti M., Puddu P.E. (2013). The pharmacological use of ellagic acid-rich pomegranate fruit. Int. J. Food Sci. Nutr..

[53] Venusova E., Kolesarova A., Horky P., Slama P. (2021). Physiological and immune functions of punicalagin. Nutrients.

[54] Yilmaz E., Arikanoğlu Z., Turkoğlu A., Kiliç E., Yüksel H., Gümüş M. (2016). The protective effects of pomegranate on liver and remote organs caused by experimental obstructive jaundice model. Eur. Rev. Med. Pharmacol. Sci..

[55] Ketnawa S., Reginio F.C., Thuengtung S., Ogawa Y. (2022). Changes in bioactive compounds and antioxidant activity of plant-based foods by gastrointestinal digestion: A review. Crit. Rev. Food Sci. Nutr..

[56] Yamini S., Paswan V.K., Shehata A.M., Choubey M., Bunkar D.S., Venkatesh V. (2023). Pomegranate (*Punica granatum* L.) seed: A review on nutritional profile, functional food properties, health benefits, and safety aspects. Ann. Phytomed..

[57] Suman M., Bhatnagar P. (2019). A review on proactive pomegranate one of the healthiest foods. Int. J. Chem. Stud..

[58] Akbari A. (2016). An overview of the characteristics and function of vitamin C in various tissues: Relying on its antioxidant function. Zahedan J. Res. Med. Sci..

[59] Silacci P., Tretola M. (2019). Pomegranate’s ellagitannins: Metabolism and mechanisms of health promoting properties. Nutr. Food Sci. Int. J..

[60] Zarfeshany A., Asgary S., Javanmard S.H. (2014). Potent health effects of pomegranate. Adv. Biomed. Res..

[61] Govoni M., Danesi F. (2022). Do Pomegranate hydrolyzable tannins and their derived metabolites provide relief in osteoarthritis? findings from a scoping review. Molecules.

[62] Li H., Ruan J., Huang J., Yang D., Yu H., Wu Y., Zhang Y., Wang T. (2023). Pomegranate (*Punica granatum* L.) and its rich ellagitannins as potential inhibitors in ulcerative colitis. Int. J. Mol. Sci..

[63] Mirjalili S.A. (2020). Pomegranate worth in women’s health—A review. Amazon. J. Plant Res..

[64] Tashkandi H.M. (2023). Therapeutic potential of pomegranate (*Punica granatum* Linn.) against breast cancer. Indian J. Pharm. Sci..

[65] Karak P. (2019). Biological activities of flavonoids: An overview. IJPSR.

[66] Prakash D., Gupta C. (2019). Phytopharmaceutical applications of nutraceutical and functional foods. Complementary and Alternative Medicine: Breakthroughs in Research and Practice.

[67] Brodowska K.M. (2017). Natural flavonoids: Classification, potential role, and application of flavonoid analogues. Eur. J. Biol. Res..

[68] Yisimayili Z., Chao Z. (2022). A review on phytochemicals, metabolic profiles and pharmacokinetics studies of the different parts (juice, seeds, peel, flowers, leaves and bark) of pomegranate (*Punica granatum* L.). Food Chem..

[69] Wang D., Özen C., Abu-Reidah I.M., Chigurupati S., Patra J.K., Horbanczuk J.O., Jóźwik A., Tzvetkov N.T., Uhrin P., Atanasov A.G. (2018). Vasculoprotective effects of pomegranate (*Punica granatum* L.). Front. Pharmacol..

[70] Vučić V., Grabež M., Trchounian A., Arsić A. (2019). Composition and potential health benefits of pomegranate: A review. Pharm. Des..

[71] Jafari T., Fallah A.A., Bahrami M., Lorigooini Z. (2020). Effects of pomegranate peel extract and vitamin E on oxidative stress and antioxidative capacity of hemodialysis patients: A randomized controlled clinical trial. J. Funct. Foods.

[72] Suručić R., Travar M., Petković M., Tubić B., Stojiljković M.P., Grabež M., Šavikin K., Zdunić G., Škrbić R. (2021). Pomegranate peel extract polyphenols attenuate the SARS-CoV-2 S-glycoprotein binding ability to ACE2 Receptor: In silico and in vitro studies. Bioorg. Chem..

[73] Ko K., Dadmohammadi Y., Abbaspourrad A. (2021). Nutritional and bioactive components of pomegranate waste used in food and cosmetic applications: A review. Foods.

[74] Masyita A., Mustika Sari R., Dwi Astuti A., Yasir B., Rahma Rumata N., Emran T.B., Nainu F., Simal-Gandara J. (2022). Terpenes and terpenoids as main bioactive compounds of essential oils, their roles in human health and potential application as natural food preservatives. Food Chem. X.

[75] Bozkurt T., Ergun Z.J.G.B., Sciences P. (2021). Fatty acid composition and antioxidant capacity of pomegranate seed oil. GSCBPS.

[76] Ge S., Duo L., Wang J., Yang J., Li Z., Tu Y. (2021). A unique understanding of traditional medicine of pomegranate, *Punica granatum* L. and its current research status. J. Ethnopharmacol..

[77] Li X., Xin Y., Mo Y., Marozik P., He T., Guo H. (2022). The bioavailability and biological activities of phytosterols as modulators of cholesterol metabolism. Molecules.

[78] Marsoul A., Ijjaali M., Elhajjaji F., Taleb M., Salim R., Boukir A. (2020). Phytochemical screening, total phenolic and flavonoid methanolic extract of pomegranate bark (*Punica granatum* L.): Evaluation of the inhibitory effect in acidic medium 1 M HCl. Mater. Today Proc..

[79] Puneeth H., Chandra S. (2020). A review on potential therapeutic properties of pomegranate (*Punica granatum* L.). Plant Sci. Today.

[80] Wong T.L., Strandberg K.R., Croley C.R., Fraser S.E., Nagulapalli Venkata K.C., Fimognari C., Sethi G., Bishayee A. (2021). Pomegranate bioactive constituents target multiple oncogenic and oncosuppressive signaling for cancer prevention and intervention. Semin. Cancer Biol..

[81] Baral S., Kaphle H.P. (2023). Health-related quality of life among menopausal women: A cross-sectional study from Pokhara, Nepal. PLoS ONE.

[82] Santoro N. (2016). Perimenopause: From research to practice. J. Womens Health.

[83] Johnson A., Roberts L., Elkins G. (2019). Complementary and alternative medicine for menopause. J. Evid. Based Integr. Med..

[84] Sussman M., Trocio J., Best C., Mirkin S., Bushmakin A.G., Yood R., Friedman M., Menzin J., Louie M. (2015). Prevalence of menopausal symptoms among mid-life women: Findings from electronic medical records. BMC Womens Health.

[85] Xue P., Zhang G., Zhang J., Ren L. (2022). Synergism of ellagic acid in combination with radiotherapy and chemotherapy for cancer treatment. Phytomedicine.

[86] Theis S., Baumgartner S.J., Janka H., Kolokythas A., Skala C., Stute P. (2023). Quality of life in menopausal women in the workplace—A systematic review. Climacteric.

[87] Minkin M.J. (2019). Menopause: Hormones, lifestyle, and optimizing aging. Obstet. Gynecol. Clin. N. Am..

[88] Moeini R., Shirafkan H., Gorji N. (2023). Pomegranate effects on the health aspects of women during peri-and postmenopause: A systematic review and meta-analysis. Phytother. Res..

[89] Newson L. (2018). Menopause and cardiovascular disease. Post Reprod. Health.

[90] Assaf A.R., Bushmakin A.G., Joyce N., Louie M.J., Flores M., Moffatt M. (2017). The relative burden of menopausal and postmenopausal symptoms versus other major conditions: A retrospective analysis of the medical expenditure panel survey data. Am. Health Drug Benefits.

[91] Wee J.H., Jung H.J., Jung K.O., Sung H.M., Shin Y.R., Park J.H., Seo H.Y., Lim J.M., Chae H.J., Lee K.Y. (2015). Pomegranate extract improves menopausal syndrome in ovariectomized rats. J. Korean Soc. Food Sci. Nutr..

[92] Mori-Okamoto J., Otawara-Hamamoto Y., Yamato H., Yoshimura H. (2004). Pomegranate extract improves a depressive state and bone properties in menopausal syndrome model ovariectomized mice. J. Ethnopharmacol..

[93] Shaban N.Z., Talaat I., Elrashidy F., Hegazy A., Sultan A. (2017). Therapeutic role of *Punica granatum* (pomegranate) seed oil extract on bone turnover and resorption induced in ovariectomized rats. J. Nutr. Health Aging.

[94] Kum E.J., Kwon D.H., Lee H. (2009). The effect of pomegranate extracts on the menopausal syndromes. J. Exp. Biomed. Sci..

[95] Kaban I., Kaban A., Tunca A.F., Aka N., Kavak H., Akar F. (2018). Effect of pomegranate extract on vagina, skeleton, metabolic and endocrine profiles in an ovariectomized rat model. J. Obstet. Gynaecol. Res..

[96] Spilmont M., Léotoing L., Davicco M.-J., Lebecque P., Mercier S., Miot-Noirault E., Pilet P., Rios L., Wittrant Y., Coxam V. (2014). Pomegranate and its derivatives can improve bone health through decreased inflammation and oxidative stress in an animal model of postmenopausal osteoporosis. Eur. J. Nutr..

[97] Lentscher J.A., Slocum B., Torrealday S. (2021). Polycystic ovarian syndrome and fertility. Clin. Obstet. Gynecol..

[98] Minocha N. (2020). Polycystic ovarian disease or polycystic ovarian syndrome: How to identify and manage—A review. Arch. Pharm. Pract..

[99] Bahmani M., Shokri S., Akhtar Z.N., Abbaszadeh S., Manouchehri A. (2022). The effect of pomegranate seed oil on human health, especially epidemiology of polycystic ovary syndrome; A systematic review. JBRA Assist. Reprod..

[100] Abasian Z., Rostamzadeh A., Mohammadi M., Hosseini M., Rafieian-Kopaei M. (2018). A review on role of medicinal plants in polycystic ovarian syndrome: Pathophysiology, neuroendocrine signaling, therapeutic status and future prospects. Middle East Fertil. Soc. J..

[101] Purwar A., Nagpure S. (2022). Insulin resistance in polycystic ovarian syndrome. Cureus.

[102] Bilal M., Haseeb A., Rehman A. (2018). Relationship of polycystic ovarian syndrome with cardiovascular risk factors. Diabetes Metab. Syndr..

[103] Barber T.M., Franks S., Leung P.C.K., Adashi E.Y. (2019). Chapter 27—Genetic and environmental factors in the etiology of polycystic ovary syndrome. The Ovary.

[104] Ajmal N., Khan S.Z., Shaikh R. (2019). Polycystic ovary syndrome (PCOS) and genetic predisposition: A review article. Eur. J. Obstet. Gynecol. Reprod. Biol. X.

[105] Wawrzkiewicz-Jałowiecka A., Kowalczyk K., Trybek P., Jarosz T., Radosz P., Setlak M., Madej P. (2020). In search of new therapeutics-molecular aspects of the PCOS pathophysiology: Genetics, hormones, metabolism and beyond. Int. J. Mol. Sci..

[106] Hossein K.J., Leila K.J., koukhdan Ebrahim T., Nazanin S.J., Farzad P., Elham R. (2015). The effect of pomegranate juice extract on hormonal changes of female Wistar rats caused by polycystic ovarian syndrome. Biomed. Pharmacol. J..

[107] Ibrahim M., Sadek M., Eldin H.S. (2022). Role of pomegranate extract in restoring endometrial androgen receptor expression, proliferation, and pinopodes in a rat model of polycystic ovary syndrome. Morphologie.

[108] Siegel R.L., Miller K.D., Wagle N.S., Jemal A. (2023). Cancer statistics, 2023. CA Cancer J. Clin..

[109] Sharma P., McClees S.F., Afaq F. (2017). Pomegranate for prevention and treatment of cancer: An update. Molecules.

[110] Waks A.G., Winer E.P. (2019). Breast cancer treatment: A review. JAMA.

[111] Britt K.L., Cuzick J., Phillips K.-A. (2020). Key steps for effective breast cancer prevention. Nat. Rev. Cancer.

[112] Newman D.J., Cragg G.M. (2007). Natural products as sources of new drugs over the last 25 years. J. Nat. Prod..

[113] Huang W.-Y., Cai Y.-Z., Zhang Y. (2009). Natural phenolic compounds from medicinal herbs and dietary plants: Potential use for cancer prevention. Nutr. Cancer.

[114] Kapoor R., Ronnenberg A., Puleo E., Chatterton R.T., Dorgan J.F., Seeram N.P., Sturgeon S.R. (2015). Effects of pomegranate juice on hormonal biomarkers of breast cancer risk. Nutr. Cancer.

[115] Khwairakpam A.D., Bordoloi D., Thakur K.K., Monisha J., Arfuso F., Sethi G., Mishra S., Kumar A.P., Kunnumakkara A.B. (2018). Possible use of *Punica granatum* (pomegranate) in cancer therapy. Pharmacol. Res..

[116] Kim N.D., Mehta R., Yu W., Neeman I., Livney T., Amichay A., Poirier D., Nicholls P., Kirby A., Jiang W. (2002). Chemopreventive and adjuvant therapeutic potential of pomegranate (*Punica granatum*) for human breast cancer. Breast Cancer Res. Treat..

[117] Adams L.S., Zhang Y., Seeram N.P., Heber D., Chen S. (2010). Pomegranate ellagitannin-derived compounds exhibit antiproliferative and antiaromatase activity in breast cancer cells in vitro. Cancer Prev. Res..

[118] Grossmann M.E., Mizuno N.K., Schuster T., Cleary M.P. (2010). Punicic acid is an omega-5 fatty acid capable of inhibiting breast cancer proliferation. Int. J. Oncol..

[119] Shirode A.B., Kovvuru P., Chittur S.V., Henning S.M., Heber D., Reliene R. (2014). Antiproliferative effects of pomegranate extract in MCF-7 breast cancer cells are associated with reduced DNA repair gene expression and induction of double strand breaks. Mol. Carcinog..

[120] Chen H.S., Bai M.H., Zhang T., Li G.D., Liu M. (2015). Ellagic acid induces cell cycle arrest and apoptosis through TGF-β/Smad3 signaling pathway in human breast cancer MCF-7 cells. Int. J. Oncol..

[121] Costantini S., Rusolo F., De Vito V., Moccia S., Picariello G., Capone F., Guerriero E., Castello G., Volpe M.G. (2014). Potential anti-inflammatory effects of the hydrophilic fraction of pomegranate (*Punica granatum* L.) seed oil on breast cancer cell lines. Molecules.

[122] Toi M., Bando H., Ramachandran C., Melnick S.J., Imai A., Fife R.S., Carr R.E., Oikawa T., Lansky E.P. (2003). Preliminary studies on the anti-angiogenic potential of pomegranate fractions in vitro and in vivo. Angiogenesis.

[123] Kim H.J., Kwon D.H., Kum E.J. (2010). Effects of *Punica granatum* (pomegranate) extracts on the menopause women. Biomed. Sci. Lett..

[124] Huber R., Gminski R., Tang T., Weinert T., Schulz S., Linke-Cordes M., Martin I., Fischer H., Tang T. (2017). Pomegranate (*Punica granatum*) seed oil for treating menopausal symptoms: An individually controlled cohort study. Altern. Ther. Health Med..

[125] Auerbach L., Rakus J., Bauer C., Gerner C., Ullmann R., Wimmer H., Huber J. (2012). Pomegranate seed oil in women with menopausal symptoms: A prospective randomized, placebo-controlled, double-blinded trial. Menopause.

[126] Adel-Mehraban M.S., Tansaz M., Mohammadi M., Yavari M. (2022). Effects of pomegranate supplement on menopausal symptoms and quality of life in menopausal women: A double-blind randomized placebo-controlled trial. Complement. Ther. Clin. Pract..

[127] Yarmohammadi M., Mahjoub P.D.S. (2017). Effects of aerobic exercise and pomegranate extract on antioxidant markers in women postmenopausal with type 2 diabetes. HMJ.

[128] (2010). Pomegranate Extract Biomarker Study in Osteopenic Women. https://classic.clinicaltrials.gov/show/NCT01219140.

[129] Sakarya U. (2024). Effect of Pomegranate Supplementation on Symptom Severity in Women with Premenstrual Syndrome. https://classic.clinicaltrials.gov/show/NCT06201702.

[130] Esmaeilinezhad Z., Babajafari S., Sohrabi Z., Eskandari M.H., Amooee S., Barati-Boldaji R. (2019). Effect of synbiotic pomegranate juice on glycemic, sex hormone profile and anthropometric indices in PCOS: A randomized, triple blind, controlled trial. Nutr. Metab. Cardiovasc. Dis..

[131] Esmaeilinezhad Z., Barati-Boldaji R., Brett N.R., de Zepetnek J.O.T., Bellissimo N., Babajafari S., Sohrabi Z. (2020). The effect of synbiotics pomegranate juice on cardiovascular risk factors in PCOS patients: A randomized, triple-blinded, controlled trial. J. Endocrinol. Investig..

[132] Abedini M., Ghasemi-Tehrani H., Tarrahi M.J., Amani R. (2021). The effect of concentrated pomegranate juice consumption on risk factors of cardiovascular diseases in women with polycystic ovary syndrome: A randomized controlled trial. Phytother. Res..

[133] Abedini M., Ramezani-Jolfaie N., Ghasemi-Tehrani H., Tarrahi M.J., Amani R. (2023). The effect of concentrated pomegranate juice on biomarkers of inflammation, oxidative stress, and sex hormones in overweight and obese women with polycystic ovary syndrome: A randomized controlled trial. Phytother. Res..

[134] Kazemi M., Lalooha F., Nooshabadi M.R., Dashti F., Kavianpour M., Haghighian H.K. (2021). Randomized double blind clinical trial evaluating the ellagic acid effects on insulin resistance, oxidative stress and sex hormones levels in women with polycystic ovarian syndrome. J. Ovarian Res..

[135] Pan L., Duan Y., Ma F., Lou L. (2020). Punicalagin inhibits the viability, migration, invasion, and EMT by regulating GOLPH3 in breast cancer cells. J. Recept. Signal Transduct. Res..

[136] Nallanthighal S., Elmaliki K.M., Reliene R. (2017). Pomegranate extract alters breast cancer stem cell properties in association with inhibition of epithelial-to-mesenchymal transition. Nutr. Cancer.

[137] Mandal A., Bhatia D., Bishayee A. (2017). Anti-inflammatory mechanism involved in pomegranate-mediated prevention of breast cancer: The role of NF-κB and Nrf2 signaling pathways. Nutrients.

[138] Bishayee A., Mandal A., Bhattacharyya P., Bhatia D. (2016). Pomegranate exerts chemoprevention of experimentally induced mammary tumorigenesis by suppression of cell proliferation and induction of apoptosis. Nutr. Cancer.

